# Morphosyntactic Abilities and Cognitive Performance in Multiple Sclerosis

**DOI:** 10.3390/brainsci14030237

**Published:** 2024-02-29

**Authors:** Panagiotis Grigoriadis, Christos Bakirtzis, Elli Nteli, Marina-Kleopatra Boziki, Maria Kotoumpa, Paschalis Theotokis, Evangelia Kesidou, Stavroula Stavrakaki

**Affiliations:** 1 School of Medicine, Aristotle University of Thessaloniki, 54124 Thessaloniki, Greece; pgrigorn@auth.gr; 2 School of Italian Language and Literature, Faculty of Philosophy, Aristotle University of Thessaloniki, 54124 Thessaloniki, Greece; 3 Multiple Sclerosis Center, Second Department of Neurology, School of Medicine, Aristotle University of Thessaloniki, 54124 Thessaloniki, Greece; cbakirtzis@auth.gr (C.B.); nteli.elli@gmail.com (E.N.); bozikim@auth.gr (M.-K.B.); ptheotokis@auth.gr (P.T.); kesidoue@auth.gr (E.K.); 4 Lab of Cognitive Neuroscience, School of Psychology, Aristotle University of Thessaloniki, 54124 Thessaloniki, Greece; marikoto@psy.auth.gr; 5 Hellenic Open University, 26335 Patra, Greece

**Keywords:** multiple sclerosis, language, cognition, morphology, syntax, memory, information processing speed

## Abstract

While cognitive abilities in people with multiple sclerosis (PwMS) have been studied in detail, little is known about linguistic abilities in PwMS and their relation to cognitive impairment. In this cross-sectional explorative study, we aim to investigate the morphosyntactic abilities of PwMS alongside their cognitive performance. Furthermore, we explore the effect of clinical factors, namely, the disease duration and MS type, on the linguistic and cognitive performance of PwMS. By so doing, we aim to shed light on neurocognitive and clinical correlates of linguistic performance in PwMS. We included 78 patients and 78 age-, sex- and education-matched healthy individuals. All participants were additionally administered the Brief International Cognitive Assessment for Multiple Sclerosis (BICAMS) battery, a verbal short-term memory task (non-word repetition) and questionnaires about mood, fatigue and quality of life. In addition, they underwent examinations with morphology and syntax tasks. PwMS were found to be impaired in morphology (past tense) and selectively impaired in syntax alongside cognitive impairments. Disease duration had the main impact on cognitive abilities. The MS type selectively impacted linguistic abilities, as shown by the remarkably deficient performance of the MS individuals with the progressive disease subtype. Linguistic impairments were predicted by only one measure of the BICAM test, namely, the Symbol Digit Modalities Test (SDMT), a measure of cognitive processing speed. Overall, this study contributes to the better understanding of the linguistic profile of PwMS by reporting selective deficits in their morphological and syntactical abilities. Furthermore, it provides insights into the clinical and cognitive correlates of linguistic performance. By so doing, it suggests clinical implications for the development of intervention programs for PwMS.

## 1. Introduction

Multiple sclerosis (MS) is a chronic disease of the Central Nervous System (CNS) that affects approximately 2.9 million people worldwide and is considered to be the most common cause of neurologic disability in young adults [1]. Diagnosis most commonly occurs between the ages of 20 and 40 years old; nevertheless, cases of pediatric and late onset MS are not rare [2]. In Greece, it is estimated that about 21,000 people are affected by MS [3].

About 50% of people with MS (PwMS) exhibit impairments in a wide variety of cognitive domains; among them, deficits in cognitive processing speed and working memory (WM) are the most frequently observed [4]. Other cognitive domains that seem to be regularly affected by MS are various other aspects of memory, attention and executive functions [5]. However, not all aspects of these cognitive functions are equally affected. Current research reveals selective impairments in WM subcomponents. In particular, while the episodic buffer was found to be severely deficient, the visual subcomponent of the visuospatial sketchpad was revealed to be preserved [6]. Furthermore, cognitive disturbances are differentially manifested in MS depending on the MS type and disease duration. Specifically, they are more often detected in PwMS with the progressive forms of the disease and in those with long disease duration [7,8]. Apparently, such deficits may expand to a social level, resulting in distress, communicational abnormalities, difficulties in the workforce and a stressful routine for those in the social environment of PwMS, especially for close relatives [9,10,11]. Despite impairments in cognitive domains and their impact on PwMS, cognitive dysfunction and neuropsychological impairment in PwMS do not account for diagnostic parameters for MS according to the revised 2017 McDonald criteria [12]. Consequently, there is a broad discussion as far as conducting routine neuropsychologic testing in these individuals is concerned. Notably, an annual cognitive assessment of stable PwMS is recommended by experts in the field [13].

Up to now, the bulk of neuropsychological research concerns the manifestation of cognitive abilities in PwMS, while the study of linguistic abilities in this population has received little attention. Consequently, linguistic deficits are not often reported. However, quite recently, some studies have investigated a wide variety of language difficulties in this population. Specifically, different domains of oral linguistic abilities have been examined by researchers in the field, such as pragmatics, syntax and morphology as well as phonology and the lexicon. Carotenuto et al. [14] provided an in-depth investigation of pragmatic abilities alongside neurocognition, social cognition, depression and fatigue. These researchers found that while pragmatic skills were severely deficient, they were not differentially manifested amongst cognitively impaired and unimpaired patients. Notably, in this study, pragmatic abilities were found to be very strongly associated with social cognition but not with depression and fatigue. Specifically, just a tendency for the association of depression and fatigue with pragmatic performance was found [14]. Furthermore, a study on the structure of language in PwMS showed grammatical and, in particular, morphological deficits in the production of subject–verb agreement, time reference/tense and grammatical aspect [15]. However, it is underlined that PwMS differed from healthy controls quantitatively and not qualitatively, as the same performance pattern was shown by all participant groups (lower performance in grammatical aspect than in subject–verb agreement and time reference). Deficient language performance in the domain of morphology was confirmed by another study, which, in addition, showed extended linguistic deficits in the domain of pragmatics and the overall structure of language (phonology, morphology and syntax) [16]. Furthermore, impairments in naming have been widely reported for PwMS [17]. In addition, impairments in phonology were confirmed by another study which has reported signs of prosodic, articulatory and resonant abnormalities in the speech production of PwMS [18]. Renauld et al. [19] performed a systematic review and reported cases of patients who show the behavioral patterns of anomia and even aphasia. However, these researchers noticed that language impairment is not a very common symptom in PwMS and suggested that the attested language impairment may be associated with general cognitive dysfunction. Another very recent review study focused on the phonology and the speech abilities of PwMS. Specifically, Plotas et al. recently performed a review study and reported that prosody, speech rate and even pronunciation were significantly affected in PwMS [20]. With respect to the written language, Kujala et al. [21] studied written language in addition to naming in two patient groups differing only in cognitive status. The results indicated that language deficiency in PwMS is associated with cognitive decline. In sum, while the overall findings regarding language abilities indicate the presence of language impairment, further research is required in this domain to investigate the extent and the nature of linguistic impairment in these patients, especially because of the scarcity of research in the field.

Exploring the manifestation of linguistic abilities in PwMS is of great significance especially for the following matter. Investigating whether deficient linguistic performance is shown in the MS condition, in which language impairment is not the primary diagnostic symptom, is significant in terms of clinical intervention. Previous research revealed linguistic impairment in clinical conditions that were not defined as primary language disorders [22] and showed the significance of employing linguistic treatment for these individuals [23]. Consequently, providing an accurate description of deficient linguistic domains can properly inform the intervention techniques applied to PwMS. In addition, in our view, the examination of the linguistic profile of PwMS in terms of its clinical and cognitive correlates offers new insights into the disease and the relation between language and cognitive abilities. First, such an approach allows for the identification of specific linguistic profiles in accordance with clinical features, for example, disease duration and MS type. By so doing, it contributes to the specificity of intervention protocols for individuals with specific MS types. Second, investigating the relation between linguistic and cognitive abilities offers insights into the architecture of the cognitive system and the (in)dependence of language abilities from linguistic abilities. Apparently, while this is a ‘hot’ and controversial issue in the field of acquired disorders [24,25], little is known in relation to PwMS [26].

While researchers in the field of MS have shown increasing interest in the manifestation of language impairment, the extent of the impairments and their association with cognitive impairments have not been clarified. In the comprehensive review paper by Renauld et al. [19], while it is recognized that deficient language usually co-occurs with cognitive disfunction, it is explicitly stated that a ‘more systematic methodological approach’ is required by future studies to reveal the specific linguistic and clinical profiles of PwMS.

Following this direction, the present study builds on previous research and, in accordance with the rationale developed above, aims to investigate the manifestation of linguistic performance in the domain of morphology and syntax in individuals with MS speaking Greek, a language different in structure from other languages where the majority of studies were conducted. As mentioned above, deficits in the structure of language in PwMS have been reported in this group, although they are not the primary symptoms of this disease; hence, the further exploration of the manifestation of linguistic symptoms is required. This manifestation is closely considered, in the present study, in relation to cognitive abilities that have been assumed to play important roles in linguistic performance, in particular, verbal/non-verbal working memory (VWM) and verbal short term memory and information processing speed [27,28,29,30,31]. Notably, PwMS were reported to show impairments in these cognitive domains, as stated above [4]. Consequently, a major hypothesis of this study is that the cognitive variables mentioned above are expected to predict linguistic performance in PwMS to some extent. In addition, if difficulties in these linguistic and cognitive variables constitute a core feature for PwMS, then they can function as a predictor for this clinical group. A further aim was to further investigate the above hypotheses for different types of MS, namely relapsing remitting MS (RRMS) and the progressive forms of MS (PMS). Finally, we aimed to investigate the overall effect of clinical factors, e.g., the type of MS and disease duration, on the linguistic/morphosyntactic and cognitive performance of PwMS, to clarify the contribution of clinical factors to the linguistic and cognitive abilities of PwMS. By so doing, the present study contributes to a better understanding of the language and cognitive abilities of PwMS, as it investigates the cognitive and clinical correlates of linguistic performance in addition to their descriptive presentation.

## 2. Materials and Methods

### 2.1. Participants

A total of 78 patients (60 or 76.9% females) with definite MS according to the 2017 revised McDonald criteria [12] were recruited from the MS Center of the Second Department of Neurology in the AHEPA University Hospital of Thessaloniki. Among them, 61 individuals (78.2%) were diagnosed with relapsing remitting MS (RRMS), 9 (11.5%) with primary progressive MS (PPMS), and 8 (10.3%) with secondary progressive MS (SPMS). With regards to the use of disease-modifying treatments (DMTs), 28 (35.8%) of the PwMS were under treatment with anti-CD20 therapy, 17 (21.7%) were receiving natalizumab, 9 (11.5%) were treated with dimethyl fumarate, 5 (6.4%) were under treatment with glatiramer acetate, 5 (6.4%) were treated with an S1P modulator, 3 (3.8%) were treated with cladribine and 11 (14.1%) did not receive any DMTs during this period. In addition, 78 healthy individuals (54 or 69.2% females), matched to age, sex and years of education, were additionally recruited. All participants fulfilled the inclusion criteria of being at least 18 years of age (adulthood), having 12 years of education (basic education) and not presenting with significant cognitive decline, psychiatric disease or substance abuse. With regards to PwMS, all participants did not present any signs of clinical or radiological inflammatory activity in the last 6 months prior to their inclusion in this study. Healthy controls were included only if they had no history of neurological/psychiatric disease and presented normal neurologic examination.

This study was performed according to the ethical standards of the Helsinki Declaration and its later amendments and was approved by the Local Ethics Committee of the Aristotle University of Thessaloniki (AUTh) (approval nr. 55723/2023). All participants provided written consent prior to their participation in this study. Each participant was tested individually, in a quiet room, with no distractions. All tests and procedures were administered in the same order; first, an assessment of physical disability was performed by an EDSS-certified neurologist (C.B.), followed by a cognitive assessment (E.N.) performed by an experienced neuropsychologist. Afterward, the linguistic tests were administered by a trainee in neurolinguistics (P.G.), under the supervision of a senior researcher in the field of neurolinguistics (S.S.). Finally, the self-administered scales were administered (M.K. and P.G.).

Participants’ demographic and clinical characteristics are presented in Table 1. In addition, the demographic and clinical characteristics of the MS subgroups, in particular, RRMS and PMS (progressive MS including PPMS and SPMS), are presented in Table 2. As expected, participants with PMS differed from participants with RRMS in age and EDSS scores [32].

### 2.2. Linguistic Tasks

All participants underwent the following linguistic tasks that focus on the morphosyntactic deficiencies of PwMS:

- Morphology: The Perfective Past Tense Test (PPTT) [33] was used to detect possible morphologic deficits. Specifically, the existing verbs subpart was employed for this study. This is an elicited production task supported by pictures, and it consists of 20 existing verbs. Among these 20 verbs, 10 are regular (sigmatic) past tense, e.g., *grafo–egrapsa* (*write–wrote*), and 10 are irregular (non-sigmatic) past tense, e.g., *pleno–eplyna* (*wash–washed*). Regular past tense is referred to as sigmatic, because the suffix -*s* is added by rule, while irregular past tense is referred to as non-sigmatic, because there is no suffix -*s*, and instead, a stem change occurs in comparison to the present stem. The examiner showed each participant a pair of two pictures. The examiner described the first picture by indicating what the person shown is doing in that picture. Then they asked the participant what the person shown in the first picture did in the second picture. The participant had to answer in past tense by using the verb they heard in the examiner’s first sentence.
- Syntax: A test consisting of 36 sentences in total was employed to detect possible syntactic deficits [34]. These 36 sentences are divided in the following groups: (a) 24 subject and object relatives, (b) 8 reversible passives and (c) 4 reflexives. The examiner showed each participant four pictures, among which only one picture matched each sentence. The examiner read each sentence aloud, and the participant then had to point to the picture which best matched the spoken sentence.

### 2.3. Cognitive Tasks

Cognitive assessment was performed with the Greek version of the Brief International Cognitive Assessment for Multiple Sclerosis (BICAMS) [35,36]. BICAMS consists of the following tests:

- Symbol Digit Modalities Test (SDMT): The SDMT assesses information processing speed [37,38]. Each participant was given a sheet with numbers and symbols that were matched to each other and was required to substitute the symbols to their numbers in 1.5 min. The oral version was used. This test has been proven to be a fast and reliable cognitive screening tool in MS [4,38].
- Greek Verbal Learning Test (GVLT): The GVLT assesses verbal learning [39]. Only the immediate recall sessions on the test are included in the BICAMS battery. According to the current literature, these sessions may assess some aspects of VWM [40]. The examiner read out a list of 16 goods that the participant should hypothetically buy from the supermarket on a Monday. The participant then had to repeat the objects listed by the examiner. The examiner listed the objects 5 times, and the participant was requested to recall them in any order an equal number of times. This task is considered to assess verbal working memory.
- Brief Visuospatial Memory Test-Revised (BVMT-R): The BVMT-R assesses visuospatial memory [41]. The examiner required the participant to look at 6 geometric shapes for 10 s and redraw them thrice as precisely as possible.

Additionally, a non-word repetition task was applied in order to assess verbal short-term memory (VSTM), which constitutes the further development of materials previously used in Greek [34,42]. The examiner read 40 non-words aloud that ranged from 3 to 6 syllables. The participant then had to repeat each non-word as precisely and quickly as possible.

### 2.4. Clinical and Other Evaluations

For the assessment of physical disability, the Expanded Disability Status Scale (EDSS) [43] was performed by an EDSS-certified neurologist. This scale is based on a rating system, ranked between 0 (no disability) and 10 (death due to MS), and is widely used in the clinical monitoring of the CNS demyelinating diseases. Disease parameters, such as disease duration and type of MS, were additionally recorded.

Since performance in cognitive testing may be influenced by fatigue and mood [4], all participants were administered the following scales:

- Modified Fatigue Impact Scale (MFIS): The MFIS assesses the impact of fatigue on daily life [44,45]. The impact of fatigue in daily functioning is quantified by a scale with a range of 0–100. Higher scores represent a higher impact of fatigue in everyday life.
- Beck Depression Inventory-Fast Screen (BDI-FS): The BDI-FS [46] is a fast self-administered screening tool for the quantification of mood and is valid for use in PwMS [47]. It consists of 7 questions, each with 3 possible answers, with a total range of 0–21. Higher scores represent worse mood disturbances.

In order to access the self-reported levels of quality of life (QoL), we administered the EuroQol-5D (EQ-5D) [48,49]. This is a 5-item questionnaire with questions regarding the impact of mobility, self-service, common activities, pain/fret and stress/depression on daily living and also includes a scale of 0–100% (Visual Analogue Scale (VAS)) about the self-reported health status at that moment. Higher scores represent better levels of quality of life.

### 2.5. Statistical Analysis

Continuous variables, namely, age and EDSS linguistic and cognitive tests’ scores, are presented as mean ± SD. Categorical variables, namely, gender and MS type, are presented as *n* (%). For the comparison of means among HC, RRMS and PMS patients, a set of ANCOVAs was conducted, with age set as a covariate, along with Bonferroni’s post hoc comparisons.

Bonferroni’s post hoc comparisons are expected to show whether there are impaired domains in PwMS compared to HCs. In addition, they are expected to show whether the linguistic and cognitive abilities of PwMS are affected by the MS subtype.

Logistic regression analysis was conducted in order to predict the relationship between groups (PwMS/HCs; dependent variable, binary) and cognitive tests (SDMT, GVLT and BVMT-R; independent variables), fatigue (MFIS; independent variable), depression (BDI), QoL (EQ-5D and VAS; independent variables) and grammar (morphology and syntax; independent variables). The analysis was conducted for all participants, as well as separately for PwMS and for HCs. Furthermore, linear regression analysis was performed to investigate the overall effect of disease duration on cognitive and linguistic task performance. In addition, linear regression analysis (stepwise method) was conducted with scores on the BICAMS measures (SDMT, GVLT, BVMT-R) as independent variables and the linguistic variables which were found to be impaired in PwMS (vs. HCs) as the dependent ones. The analysis was conducted on IBM SPSS 27.0 and 29.0 software.

## 3. Results

### 3.1. Between Group Comparisons I: PwMS vs. HCs

With regards to cognitive testing, PwMS exhibited statistically significantly worse performance in SDMT and BVMT-R (*p* = 0.001) and VSTM (*p* < 0.001), but not in GVLT (*p* = 0.4), compared to the HCs. With regards to the linguistic assessment, PwMS performed worse in PPTT (total score *p* < 0.001, regular and irregular past tense *p* < 0.001). As far as receptive syntax is concerned, the performance of PwMS was significantly worse only in passives (*p* = 0.02). In addition, the performance of PwMS in questionnaires assessing mood, fatigue and QoL exhibited statistically significant differences (*p* = 0.01, *p* = 0.01, *p* = 0.002 respectively) compared to HCs. The performances in all tests and questionnaires of each study group are presented in Table 3. Overall, in the linguistic domain, the findings indicate a significant impairment only in passives, while they show a relatively massive impairment in the cognitive domain.

### 3.2. Between Group Comparisons II: HC, RRMS, and PMS Patients

Upon the mean comparison of cognitive scales’ scores, self-reported fatigue and QoL metrics between PwMS with RRMS vs. PMS (progressive disease; SPMS and PPMS combined in one group), a statistically significant difference was observed in EQ-5D (*p* < 0.001). With regards to the performance in linguistic tests, RRMS patients performed significantly better in the syntax test (total score) (*p* = 0.028) and object relatives (*p* < 0.001) than those with progressive MS (PMS). The overall findings indicate significant vulnerability in the PMS subgroup compared to the RRMS group in the domain of syntax (overall score and object relatives).

With respect to the comparisons of RRMS and PMS with HC in cognitive scales’ scores and self-reported fatigue and QoL metrics, it appears that PMS but not RRMS performed significantly lower than HC in SDMT (*p* = 0.003), BDI-FS (*p* = 0.031), MFIS (*p* = 0.031) and VAS (*p* < 0.001). With respect to the comparisons of RRMS and PMS with HC in linguistic tests, it appears that PMS but not RRMS performed significantly lower than HC in syntax (total score) (*p* = 0.007), object relatives (*p* < 0.001) and passives (*p* = 0.029). In addition, comparisons between RRMS and PMS and HC indicated that RRMS but not PMS performed significantly below HC in the linguistic measures of PPTT (total score) and regular and irregular past tense (*p* = 0.001, *p* < 0.001, and *p* = 0.003, respectively). The performances in all tests and questionnaires of each study group are presented in Table 4.

### 3.3. Regression Analysis I: Logistic Regression

With respect to regression analysis, cognitive tests collectively strongly predicted participants’ group (PwMS vs. HCs) (SDMT: B = −0.038; GVLT: B = 0.023; BVMT-R: B = −0.96; R^2^ = 0.16, *p* < 0.001). Within the model, SDMT and BVMT-R predicted participants’ group (*p* = 0.04 and *p* = 0.006, respectively). Fatigue predicted participants’ group (B = 0.027; R^2^ = 0.056, *p* = 0.01). QoL variables collectively strongly predicted participants’ group (EQ-5D: B = 0.488; VAS: B = −0.004; R^2^ = 0.186, *p* < 0.001). Within the model, EQ-5D mainly predicted participants’ group (*p* < 0.001). Cognitive tests, fatigue and QoL metrics collectively strongly predicted participants’ group (SDMT: B = −0.023; GVLT: B = 0.024; BVMT-R: B = −0.98; MFIS: B = −0.007; EQ-5D: B = 0.511; VAS: B = 0.003; R^2^ = 0.273, *p* < 0.001). Within the model, BVMT-R and EQ-5D predicted participants’ group (*p* = 0.008; *p* = 0.001, respectively). Syntax collectively did not predict participants’ group. The BDI also predicted participants’ group (R^2^ = 0.058, *p* = 0.009). However, when syntax metrics were considered separately as independent variables in the model, the variable ‘passives’ was able to predict the group (B = −0.332; R^2^ = 0.045, *p* = 0.021). Morphology metrics collectively were able to predict participants’ group (regular past: B = −0.247; irregular past: B = −0.039; R^2^ = 0.142, *p* < 0.001). Moreover, when morphology metrics were considered separately as independent variables in the model, the total correct score, the regular past tense correct score and the irregular past tense correct score were able to predict participants’ group (B = −0.141; R^2^ = 0.137, *p* < 0.001; B = −0.281; R^2^ = 0.141, *p* < 0.001; B = −0.242; R^2^ = 0.116, *p* < 0.001, respectively). In addition, VSTM score was able to predict participants’ group (B = −0.134; R^2^ = 0.168, *p* < 0.001).

To summarize, in addition to the significance of fatigue and QoL measures, these data reveal the contribution of cognitive measures in the identification of PwMS. Furthermore, the morphology metrics as well as performance on passives (subpart of syntax) were found to have predictive value for PwMS identification.

Upon sub-group analysis, with patients with RRMS (N = 62) and patients with progressive MS (N = 16) being regarded as separate groups, cognitive tests collectively were able to predict subgroup (SDMT: B = −0.073; GVLT: B = 0.028; BVMT-R: B = −0.063; R^2^ = 0.185, *p* < 0.02). Within the model, SDMT predicted subgroup (*p* = 0.024). Fatigue exhibited a tendency to predict subgroup (B = 0.03; R^2^ = 0.068, *p* = 0.063). QoL variables collectively strongly predicted subgroup (EQ-5D B = 0.757; VAS B = −0.038; R^2^ = 0.481, *p* < 0.001). Within the model, EQ-5D mainly predicted subgroup (*p* = 0.002), whereas VAS exhibited a tendency to predict subgroup (*p* = 0.057). Cognitive tests, fatigue and QoL metrics collectively strongly predicted subgroup (SDMT: B = −0.081; GVLT: B = 0.085; BVMT-R: B = −0.13; MFIS: B = −0.079; EQ-5D: B = 1.3; VAS: B = −0.043; R^2^ = 0.623, *p* < 0.001). Within the model, MFIS and EQ-5D predicted subgroup (*p* = 0.03 and *p* = 0.001, respectively), whereas SDMT did not predict group (*p* = 0.095). The syntactic test collectively strongly predicted subgroup (total score: B = 1.660; subject relatives: B = −1.422; object relatives: B = −2.601; passive sentences: B = −1.754; R^2^ = 0.335, *p* < 0.001). Within the model, object relatives predicted subgroup (*p* = 0.011), whereas the overall syntactic score and performance in passives exhibited a tendency to predict subgroup (*p* = 0.057 and *p* = 0.068, respectively). Morphology metrics collectively were not able to predict subgroup (R^2^ = 0.011, *p* = 0.767). Also, when morphology metrics were considered separately as independent variables in the model, neither metric was able to predict subgroup. VSTM score was able to predict participants’ group (B = −0.106; R^2^ = 0.114, *p* = 0.015). Of note, within the group of PwMS, 16 patients with PMS were included, as well as 62 patients with RRMS. Due to the fact that patients with PMS are significantly under-represented in the group of PwMS, the results of the sub-group analysis should be interpreted with caution.

To summarize, the subgroup patient analysis revealed that in addition to fatigue and quality of life measures that were successful predictors for the patient subgroup, SDMT functioned as the best cognitive subgroup predictor. While morphology metrics failed to predict the patient subgroup, object relatives (syntactic variable) were the most successful to do so. In addition, the non-word repetition task (VSTM) was revealed as a significant subgroup predictor.

### 3.4. Regression Analysis II: Linear Regression

Linear regression analysis was performed to investigate the overall effect of disease duration on performance in cognitive and linguistic tasks. We found that disease duration was a significant predictor for patient performance on the Verbal Short Term Memory Test (VSTM) (B = 0.325, *p* = 0.001, R^2^ = 0.13) and on the Brief Visuospatial Memory Test-Revised (BVMT-R) (B = 0.227, *p* = 0.031, R^2^ = 0.06). However, disease duration was not a significant predictor for SDMT (B = 0.141, *p* = 0.454, R^2^ = 0.007), GVLT (B = 0.179, *p* = 0.353, R^2^ = 0.011), syntax (total score) (B = 0.001, *p* = 0.993, R^2^ = 0.001), subject relatives (B = 0.005, *p* = 0.792, R^2^ = 0.001), object relatives (B = 0.014, *p* = 0.613, R^2^ = 0.003), passives (B = 0.002, *p* = 0.928, R^2^ = 0.10), reflexives (B = 0.008, *p* = 0.314, R^2^ = 0.013), past tense (total score) (B = 0.034, *p* = 0.749, R^2^ = 0.001), regular past (B = 0.026, *p* = 0.646, R^2^ = 0.003) or irregular past (B = 0.008, *p* = 0.881, R^2^ = 0.000). These findings indicate that disease duration significantly impacts impairment in verbal and visuospatial memory in PwMS.

In addition, we performed linear regression analysis (stepwise method) with scores on the BICAMS measures (SDMT, GVLT and BVMT-R) as independent variables and the linguistic variables *past tense (overall score), regular and irregular past tense* and *passives* as the dependent ones. By so doing, we aimed to investigate whether the attested vulnerability in *past tense (overall score), regular and irregular past tense* and *passives*, as shown by the between group (PwMS vs. HC) analysis (Table 2), can be predicted by the BICAMS cognitive measures. In our view, it is of particular interest to assess the sensitivity of BICAMS measures in predicting language impairment in MS, as BICAMS measures are widely used in disease diagnosis and considered as the representative indices (especially the information processing speed and aspects of working memory) of cognitive impairment observed in PwMS [5,35]. Only the information processing speed measure (SDMT) was found to be a significant predictor for PwMS performance on past tense (overall score) (B = 0.196, *p* = 0.002, R^2^ = 0.122), regular past tense (B = 0.117, *p* < 0.001, R^2^ = 0.392), irregular paste tense (B = 0.079, *p* = 0.013, R^2^ = 0.079) and passive sentences (B = 0.046, *p* < 0.001, R^2^ = 0.163). By contrast, GVLT and BVMT-R were found to be excluded variables for past tense (overall score) (B = 0.039, p = 0.741 and B = 0.043, *p* = 0.721, respectively), regular past tense (B = 0.036, *p* = 0.757 and B = 0.086, *p* = 0.463, respectively), irregular past tense (B = 0.040, *p* = 0.742 and B = 0.006, *p* = 0.960, respectively) and passives (B = 0.078, *p* = 0.502 and B = 0.014, *p* = 0.905, respectively). By contrast, GVLT and BVMT-R were found to be excluded variables for past tense (overall score) (B = 0.039, *p* = 0.741 and B = 0.043, *p* = 0.721, respectively), regular past tense (*B* = 0.036, *p* = 0.757 and *B* = 0.086, *p* = 0.463, respectively), irregular past tense (B = 0.040, *p* = 0.742 and B = 0.006, *p* = 0.960, respectively) and passives (B = 0.045, *p* = 0.786 and B = 0.052, *p* = 0.774, respectively). These findings indicate that abilities related to information processing speed, which are assessed by SDMT, are strongly correlated with core morphosyntactic impairments in PwMS.

## 4. Discussion

This study was set up to investigate the specific morphosyntactic abilities of PwMS alongside their cognitive abilities. It also took into account the effect of clinical factors on the performance of PwMS, in particular, the disease duration and the MS subtype. Furthermore, it evaluated the impact of cognitive variables on the PwMS linguistic performance to shed light on the cognitive and linguistic profile of PwMS.

With respect to the performance of PwMS on the cognitive measures, it should be pointed out that PwMS exhibited worse performance in information processing speed (SDMT) and visuospatial memory (BVMT-R) than HCs. Further analysis revealed that people with PMS and not RRMS performed significantly worse in the SDMT when compared to HCs, in line with previous studies that demonstrated that information processing speed is notably affected in the progressive forms of the disease [50,51]. In agreement with these findings, the logistic regression analysis revealed that the SDMT was the best cognitive subgroup predictor. The performance of PwMS in VWM, as measured by GVLT, did not differ from the performance of HCs. The fact that SDMT and BVMT-R are more capable than GVLT in detecting deficits in cognitive performance among PwMS has also been demonstrated in relevant studies [52,53]. In BVMT-R, participants are required not only to memorize geometric shapes but also to redraw them in the same order and position. Verbal memory tests, such as the immediate recall part of the GVLT that is used in the BICAMS, are more flexible in this regard, because they allow the repetition of the words read aloud by the examiner in any order and without time restriction [54,55]. In addition, there is no motor component that could interfere with performance in the GVLT.

Not surprisingly, PwMS showed deficient performance on the non-word repetition task, indicating impairments in the domain of VSTM for this population. In fact, VSTM testing was conducted as a form of additional assessment of verbal memory. Remarkably, while PwMS did not show deficient performance on GVLT, which is based on existing words, they did show deficits in non-word repetition. This finding underlines the selective way in which impairments in verbal memory are manifested in PwMS. In agreement with these findings, VSTM score could predict the patients’ subgroup indicating the significant disease type effect on the VSTM abilities of individuals with PwMS. Although VSTM and GVLT are considered to address memory aspects, there is a difference as far as the time and cognitive strategy are concerned [56]. Apparently, non-word repetition may require stronger memory abilities than existing word repetition. This is so because existing words, which belong to common groups, are easier to retrieve and pronounce [57]. Additionally, naming errors and dysarthria, which is often observed in this population [58,59], may also contribute to the worse performance in non-word repetition.

As far as the linguistic abilities of PwMS are concerned, it was found that PwMS exhibit vulnerability in linguistic abilities, specifically through morphosyntactic abnormalities, along with deficits in cognitive abilities, such as verbal short-term memory and information processing speed. In particular, in this study, PwMS performed significantly worse in the past tense morphology assessment, as compared to HCs. This finding indicates a morphological deficit in MS, which affects abilities in both regular and irregular past tense production. While the impairments in irregular past tense production can be related to the reduced verbal memory abilities of this population, which affect form retrieval, the observed deficit in the regular past tense production indicates an apparent grammatical impairment [60]. Further evidence for grammatical impairment in PwMS comes from their deficient performance in passive sentences when compared to HCs. This is presumably due to the fact that passive sentence formation requires complex syntactic processes, namely, the object of the active voice turns into the subject of the passive voice (a process known as A-movement in linguistics) [61]. Remarkably, the individuals with PMS performed significantly below those with RRMS in syntax (overall score) and object relatives. In object relatives, complex syntactic processes, i.e., syntactic movement (a process known as A-bar movement in linguistics) take place, as the object phrase undergoes movement resulting in a word orders that is different from subject verb object, unlike subject relatives [62]. Not surprisingly, as the logistic regression analysis revealed, object relatives functioned as the most successful predictor (amongst the syntactic measures) for patient subgroup identification. It seems that neurogenerative processes, most commonly seen in progressive forms of the disease (individuals with PMS in this study), may affect complex syntactic abilities, which are required for structures with syntactic movement. Additional evidence for a syntactic deficit in PMS comes from the comparisons of RRMS and PMS with HCs, which show that people with PMS but not RRMS performed significantly lower than HCs in syntax (total score), object relatives and passives. This means that the linguistic deficit appears mainly at the sentence level (syntax) for these patients. On the other hand, comparisons between RRMS and PMS and HCs indicated that people with RRMS but not PMS performed significantly below HCs in the linguistic measures of PPTT (past tense morphology; total score) and regular and irregular past tense. It should be pointed out that a failure to find significance (*p* almost equal to 0.05 for the total score-past tense and the regular past tense) for the comparison between HCs and people with PPS may be related to the small number of participants with PPS. However, the *p* value indicating non-significance between HCs and PMS is rather stronger for the irregular past tense (*p* = 0.079). It seems that a deficit at the word level (especially irregular morphology) is less evident in people with PMS than those with RRMS. While we are aware of the limitation concerning the small number of people with PMS, we suggest, based on the available data, that people with PPS show difficulties especially in the syntactic component of language, and people with RRMS show difficulties at the word level. Collectively, these data show impairments in grammar (morphology and syntax), a finding which fits well with recent findings concerning the linguistic abilities of PwMS [56].

In addition, our study revealed that disease duration affects cognitive domains as it has a significant impact on impairment in VSTM and visuospatial memory in PwMS. Furthermore, as the logistic regression analysis revealed, the participant group was predicted by SDMT, BVMT-R, the past tense score and the passives sub-score, indicating the significance of those measures in PwMS identification. Remarkably, the only cognitive measure that was found to be a significant predictor for PwMS performance on those linguistic variables that was impaired in PwMS compared to HCs was the SDMT subtest. It should be pointed out that this measure assesses cognitive processing speed. In addition, it is widely employed in clinical practice for PwMS. Furthermore, it was found to appropriately capture cognitive dysfunction in MS [63]. Crucially, it predicts linguistic performance, and it is, thus, suggested that deficits in cognitive processing speed, as revealed by the SDMT subtest, seem to be closely connected with linguistic decay in PwMS.

To the best of our knowledge, the predictive value of cognitive processing speed, as measured by SDMT, for the linguistic performance of PwMS has not been reported in the relevant MS literature. Therefore, further explorations in this domain are needed. In particular, intervention studies that could provide therapy in the domain of cognitive processing speed and assess whether therapy effects could transfer to language and especially complex syntax would be very informative, in our view. Alternatively, it is of great importance to investigate whether cognitive abilities are positively affected by linguistic training in this population. So far, preserved cognitive performance in bilinguals with MS [64] has been reported, a finding which may indicate that linguistically oriented intervention may be beneficial for PwMS.

Apparently, there are some limitations in this study. In particular, we did not include the type of DMTs used in the analysis, since according to the current literature, DMTs have no or minimal effect on cognitive functions [4,65,66]. Furthermore, the linguistic tests employed in this study have been used in previous studies with Greek-speaking populations but were not published per se; hence, comparisons can be only made with reference to the mean performance of healthy controls. Another limitation is related to the small number of participants with PPS. In addition, we only tested information processing speed and some aspects of working memory as potential predictors of linguistic impairment. We selected the above-mentioned cognitive domains because they represent a pattern of cognitive impairment that is observed in PwMS [5,35]. Additionally, several studies have demonstrated that these cognitive functions may interact with linguistic abilities, while they may share common neural circuits [67,68,69]. Nevertheless, a more detailed neuropsychological assessment, including the use of the neuroimaging of linguistic and cognitive performances, might reveal correlations between other cognitive functions and linguistic deficits, and it is worth further exploration. Furthermore, evaluators were not blinded to the study population. In addition, this study had a cross-sectional design; therefore, longitudinal observations of linguistic and cognitive abilities were not performed. Finally, within the frame of this study, additional analyses in order to directly statistically compare the statistical models with cognitive abilities and linguistic performance were not performed.

In conclusion, this study highlighted the deficits in PwMS in the linguistic domain alongside their cognitive impairment. It revealed the correlations between clinical measures and linguistic impairment by characterizing the deterioration of crucial syntactic abilities in the progressive form of the disease. In addition, it showed that linguistic impairments were best predicted by the SDMT, a measure of cognitive processing speed. We claim that these findings have clinical implications and point to fruitful ways for providing intervention in PwMS by including training in cognitive processing speed and exploring its effects on linguistic abilities.

## Figures and Tables

**Table 1:** Demographic and clinical characteristics of the study population.

	PwMS	HCs	*p*-Value
*n*	78	78	
Females, *n* (%)	60 (76.9%)	54 (69.2%)	0.28
Age (mean, SD)	37.5 ± 9.8	35.7 ± 11.3	0.28
Education (years)	14.9 ± 2.3	15.4 ± 2.3	0.19
12 (*n*, %)	24 (30.8%)	19 (24.3%)	0.4
13–16 (*n*, %)	30 (38.5%)	30 (38.5%)	1.0
17+ (*n*, %)	24 (30.8%)	29 (37.2%)	0.4
MS disease characteristics			
Disease duration (mean, SD)	9.2 ± 6.9		
EDSS (median, min–max)	2.0 (1–7.5)		
Type of MS			
RRMS (*n*, %)	61 (78.2%)		
SPMS (*n*, %)	8 (10.3%)		
PPMS (*n*, %)	9 (11.5%)		

Note: PwMS: people with multiple sclerosis, HCs: healthy controls, MS: multiple sclerosis, SD: standard deviation, EDSS: Expanded Disability Status Scale, RRMS: relapsing remitting multiple sclerosis, SPMS: secondary progressive multiple sclerosis, PPMS: primary progressive multiple sclerosis.

**Table 2:** Demographic and clinical characteristics of the MS subgroups.

	RRMS	PMS	*p*-Value
*n*	61	17	
Females, *n* (%)	62	16	0.20
Age (mean, SD)	35.6 ± 9.6	44.9 ± 7.6	<0.001
Education (years)	15 ± 2.4	14.6 ± 1.96	0.52
MS disease characteristics			
Disease duration (mean, SD)	8.72 ± 6.60	11.75 ± 7.73	0.12
EDSS (median, min-max)	2.22 (1–6)	5.47 (3.5–7.5)	<0.001
Currently under DMT	59 (96.7%)	8 (35.2%)	<0.001

Note: MS: multiple sclerosis, SD: standard deviation, EDSS: Expanded Disability Status Scale, RRMS: relapsing remitting multiple sclerosis, PMS: progressive multiple sclerosis, DMT: disease-modifying treatment.

**Table 3:** Performance in tests and questionnaires of each study group.

	PwMS, *n*: 78 (MEAN, SD)	HCs, *n*: 78 (MEAN, SD)	*p*-Value
BICAMS			
SDMT	50.5 ± 11.2	56.6 ± 11.5	0.001
GVLT	56.9 ± 11.5	58.5 ± 11.2	0.4
BVMT-R	27.3 ± 6.3	31.0 ± 5.3	<0.001
VSTM			
Total score	26.0 ± 6.2	30.4 ± 5.1	<0.001
MORPHOLOGY TEST (PPTT)			
Total score	14.2 ± 6.3	17.6 ± 3.4	<0.001
Regular	7.4 ± 3.3	9.2 ± 1.7	<0.001
Irregular	6.8 ± 3.1	8.4 ± 1.9	<0.001
SYNTAX TEST			
Total score	31.7 ± 3.5	32.7 ± 3.2	0.8
Subject relatives	7.0 ± 1.0	7.3 ± 1.1	0.2
Object relatives	14.1 ± 1.6	14.5 ± 1.7	0.3
Passives	6.7 ± 1.2	7.1 ± 1.0	0.02
Reflexives	3.7 ± 0.4	3.8 ± 0.4	0.2
BDI-FS	3.3 ± 3.4	2.2 ± 1.9	0.01
MFIS	26.8 ± 17.1	20.8 ± 13.1	0.01
EQ-5D			
5 items score	7.4 ± 1.8	6.2 ± 1.3	<0.001
VAS	75.5 ± 18.3	83.5 ± 13.2	0.002

Note: PwMS: people with multiple sclerosis, HCs: healthy controls SD: standard deviation, BICAMS: Brief International Assessment for Multiple Sclerosis, SDMT: Symbol Digit Modalities Test, GVLT: Greek Verbal Learning Test, BVMT-R: Brief Visuospatial Memory Test-Revised, VSTM: Verbal Short-Term Memory, PPTT: Perfective Past Tense Test, BDI-FS: Beck Depression Inventory-Fast Screen, MFIS: Modified Fatigue Impact Scale, EQ-5D: EuroQol-5D, VAS: Visual Analogue Scale.

**Table 4:** Performance in tests and questionnaires of HC, RRMS and PMS patients.

	HC, *n*: 78 (Mean, SD)	RRMS, *n*: 61 (Mean, SD)	PMS, *n*: 17 (Mean, SD)	*p*-Value (ANCOVA)	*p*-Value (Bonferroni’s Post Hoc) *
BICAMS					
SDMT	56.6 ± 11.5	52.4 ± 10.1	43.3 ± 12.7	0.001	0.06; 0.003; 0.156
GVLT	58.5 ± 11.2	57.6 ± 11.6	54.6 ± 11.0	0.611	N/A
BVMT-R	31.0 ± 5.3	28.1 ± 5.8	24.4 ± 7.5	<0.001	0.008; 0.003; 0.417
VSTM					
Total score	30.4 ± 5.1	27.0 ± 6.0	22.6 ± 6.0	<0.001	<0.001; <0.001; 0.21
PPTT					
Total score	17.6 ± 3.4	14.5 ± 6.2	13.2 ± 6.5	<0.001	0.001; 0.054; 1
Regular	9.2 ± 1.7	7.5 ± 3.4	6.9 ± 3.3	<0.001	<0.001; 0.056; 1
Irregular	8.4 ± 1.9	7.0 ± 3.1	6.3 ± 3.5	<0.001	0.003; 0.079; 1
SYNTAX TEST					
Total score	32.7 ± 3.2	32.3 ± 3.4	29.6 ± 3.4	0.009	1; 0.007; 0.028
Subject relatives	7.3 ± 1.1	7.1 ± 1.1	6.8 ± 1.0	0.305	N/A
Object relatives	14.5 ± 1.7	14.5 ± 1.5	12.8 ± 1.7	<0.001	1; <0.001; <0.001
Passives	7.1 ± 1.0	6.8 ± 1.2	6.1 ± 1.5	0.026	0.448; 0.029; 0.264
Reflexives	3.8 ± 0.4	3.8 ± 0.5	3.8 ± 0.4	0.631	N/A
BDI-FS	2.2 ± 1.9	3.2 ± 3.3	4.3 ± 3.7	0.016	0.159; 0.031; 0.487
MFIS	20.8 ± 13.1	25.0 ± 17.2	34.0 ± 14.6	0.02	0.227; 0.031; 0.413
EQ-5D					
5 items score	6.2 ± 1.3	7.0 ± 1.6	9.5 ± 1.4	<0.001	0.006; <0.001; <0.001
VAS	83.5 ± 13.2	79.7 ± 16.2	58.7 ± 16.4	<0.001	0.415; <0.001; <0.001

Note HC: healthy controls; RRMS: relapsing remitting multiple sclerosis, PMS: progressive multiple sclerosis, SD: standard deviation, BICAMS: Brief International Assessment for Multiple Sclerosis, SDMT: Symbol Digit Modalities Test, GVLT: Greek Verbal Learning Test, BVMT-R: Brief Visuospatial Memory Test-Revised, VSTM: Verbal Short-Term Memory, PPTT: Perfective Past Tense Test, BDI-FS: Beck Depression Inventory-Fast Screen, MFIS: Modified Fatigue Impact Scale, EQ-5D: EuroQol-5D, VAS: Visual Analogue Scale, ANCOVA: analysis of covariance, N/A: not applicable. * For Bonferroni’s post hoc comparisons, *p* values represent the following: healthy controls vs. relapsing remitting multiple sclerosis; healthy controls vs. progressive multiple sclerosis; relapsing remitting multiple sclerosis vs. progressive multiple sclerosis.

## Data Availability

The data presented in this study are available upon request from the corresponding author. The data are not publicly available due to ethical reasons.
